# Selenol Protecting Groups in Organic Chemistry: Special Emphasis on Selenocysteine Se-Protection in Solid Phase Peptide Synthesis

**DOI:** 10.3390/molecules16043232

**Published:** 2011-04-18

**Authors:** Stevenson Flemer

**Affiliations:** 116 Cook Bldg; 82 University Place; University of Vermont; Burlington, VT 05405, USA; E-mail: sflemer@uvm.edu; Tel.: +1-802-656-0269; Fax: +1-802-656-8705.

**Keywords:** selenium, selenocysteine, protecting group, protection

## Abstract

The appearance of selenium in organic synthesis is relatively rare, and thus examples in the literature pertaining to the masking of its considerable reactivity are similarly uncommon. Greene's Protecting Groups in Organic Synthesis, the standard reference for the state of the art in this arena, offers no entries for selenium protective methodology, in stark comparison to its mention of the great variety of protecting groups germane to its chalcogen cousin sulfur. This scarcity of Se-protection methods makes it no less interesting and pertinent toward the construction of selenium-containing organic systems which do indeed require the iterative blocking and de-blocking of selenol functionalities. A selenium-containing system which is especially relevant is selenocysteine, as its use in Solid Phase Peptide Synthesis requires extensive protection of its selenol side chain. This review will attempt to summarize the current state of understanding with regard to selenium protection protocol in organic synthesis. Moreover, it will provide a special emphasis on selenocysteine side chain protection, comprising both the breadth of functionality used for this purpose as well as methods of deprotection.

## 1. Introduction

**Figure 1:**
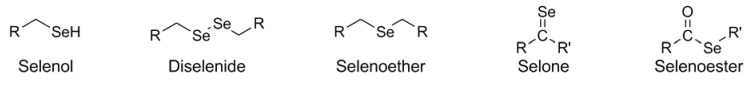
Various functional groups containing selenium and their nomenclature.

Selenium is a chalcogen element which, although technically a non-metal, is frequently referred to as "selenium metal" in industrial parlance and MSDS identification vernacular. Chemically related to sulfur and oxygen, selenium has a wide variety of utilities in the inorganic arena, including uses in semiconductors, photovoltaic and photocell devices, and photographic toner applications. It has prime industrial uses in the glass and ceramic industry to produce deep red coloring in these materials. Moreover, elemental selenium is an important biological micronutrient, essential to human health. In contrast to the wide variety of inorganic selenium application, organic selenium (*i.e.:* in compounds bearing carbon-selenium covalent bonds) occupy a singular niche within the overall realm of selenium chemistry. Although the appearance of selenium in organic synthesis is relatively rare in comparison with the breadth of applications attributable to its chalcogen cousins, it does figure prominently in many organic transformations, whether used as a component of the reagent in a chemical reaction or the organic substrate upon which it is acting. Virtually all organic structural motifs which are possible with oxygen and sulfur are feasible via isosteric replacement with selenium, although the practical application of selenium-based functional grouping is not always straightforward or pertinent. Figure 1 illustrates some of the existing Se-based functional groups.

**Figure 2:**
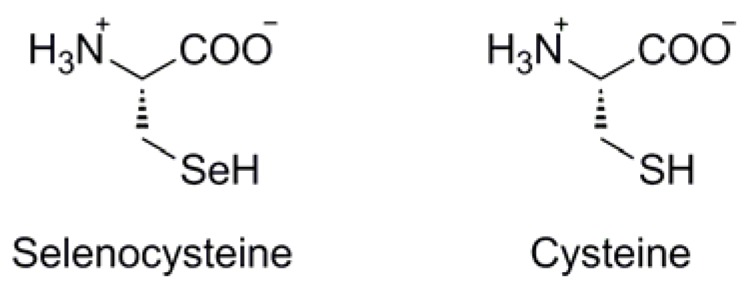
Structural comparison between selenocysteine and cysteine.

In its selenol form (ie: R-SeH), organic selenium is at its most reactive. Due to its great difference in pKa compared with that of sulfur (ie: pKa ~5 for selenol vs ~8 for thiol), the selenol functionality in biological systems will exist as the corresponding selenoate (ie: R-Se^-^), acting as a strong nucleophile with high oxidative potential. Indeed, it is within biological systems that scientific interest in selenium is at its maximum. The most prevalent source of selenium within biological systems is the amino acid selenocysteine (Sec, U), in which the amino acid sidechain is isosteric with cysteine, but bearing a selenol functionality rather than a thiol (Figure 2). The Sec selenol is a crucial component of many important enzymatic redox pathways such as those mediated by thioredoxin reductase [1] and glutathione peroxidase [2], wherein the iterative formation and subsequent reduction of selenylsulfide structures within the Sec/Cys framework of the enzymes mediate electron flow to and/or from the enzyme's substrate, dependent upon it's redox function. 

In chemical as well as biological systems, the reactivity of the selenol functionality can be a mixed blessing. While a synthetic target may bear reactive selenol architecture specific to its function, this reactivity must typically be attenuated or blocked while the compound is being synthesized in order to avoid unwanted side reactions attributable to this reactive center. This attenuation of reactive functionality is usually accomplished through the use of protecting groups (organic scaffolding which can be carried through the steps of construction), but which can be removed at the end of the synthesis, to regenerate the native functionality. Surprisingly, there is a striking scarcity of existing protection protocol for the selenol functionality in comparison with that available for its analogous chalcogen analog, the thiol. Indeed, Greene's "Protective Groups in Organic Synthesis" [3], the prime reference in this field, while listing 84 different types of protection protocol for the thiol, has no entries whatsoever for the selenol functionality. Since there is no standard of reference for selenol protection, a review of the state of existing selenol protection protocol is warranted.

This review will comment on the present state of knowledge with regard to selenol protection protocols in organic synthesis, summarizing each type of protection motif based upon its underlying carbon architecture. Table 1 graphically illustrates the range of known selenol protection, specifying the methods of introduction as well as deprotection conditions for each functionality, citing specific references for each transformation. Specific examples of the uses of each type of protection scheme will be included whenever possible, with commentary as to the pertinence of each blocking motif within the organic system in which it is being used. A significant amount of discussion in this review will be centralized around the richer history of Se-protection protocol for selenocysteine during its incorporation into peptide systems in Solid Phase Peptide Synthesis (SPPS). It should be mentioned that, due to the remarkable lack of standard Se protection example, the term "protecting group" is used somewhat loosely in many examples given here in order that a complete listing of potential architecture be realized. 

**Table 1:** Known selenol protection schemes.

Name	Structure	Method of Introduction	Ref	DeprotectionConditions	Ref
Diselenide (**1**)	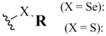	RSe- with RS-X (Se-S)	6,7,8	NaBH_4 _(Se-Se)	12
				SO_2_Cl_2_ (Se-Se)	13
Selenylsulfide (**2**)				DTT (Se-S)	14
Thiuocyanate (**3**)	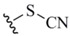	KSCN nucleophile	15	- - -	- -
Cyano (**4**)	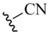	KSeCN nucleophile	17,18	NaBH_4_/LiBEt_3_H	21,22
		Me_3_SiCN nucleophile			
			13	KOH	19
2-Cyanoethyl (**5**)	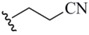	NC(CH_2_)_2_Se nucleophile	25,26	K_2_CO_3_/MeOH	25,26
		NC(CH_2_)_2_Se phthalamide	27	DBU	27
Acetate (**6**)	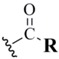	AcCl electrophile	29	NH_4_OH/THF	29
			32	KOH/MeOH-	
		KSeAc nucleophile			
[R=CH_3_]					
					32
		RSeCN/Bu_3_P-RCOOH			
			28	DCM	
Carbonate (**7**)		ClCO_2_R electrophile	29	NH_4_OH/THF	29
[R=OR]					
Carbamate (**8**)		ClCONR_2 _electrophile	29	NaOH/THF-MeOH	29
[R=NR_2_]					
Acetoxymethyl (**9**)	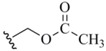	RSe(O)CH_3_/AcOH	33	H_2_O_2_	33
Phthalimide (**10**)	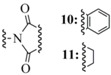	Potassium phthalimide nucleophile	34	- - -	- -
Succinimide (**11**)		N-Chloro Succinimide electrophile	36	- - -	- -
Methyl (**12**)	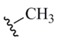	Methyl electrophile	13,38	Br_2_	41
		(CH_3_Se)_2_ electrophile	39		
		CH_3_Se^-^ nucleophile	40		
Allyl (**13**)	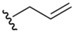	Allyl electrophile	42,43	m-CPBA/NH_2_NH_2_	42,43
Phenyl (**14**)	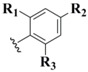	Enolate α- selenation	45	H_2_O_2_	46
		PhSeX electrophile	46,47	NaIO_4_	50
		PhSe^-^ nucleophile	48,49	O_3_	47
[R_1_,R_2_,R_3_=H]					
2,4,6-tri-*tert*-Butylphenyl (**15**)		ArSe nucleophile	54,55	Bu_3_SnH/AIBN	54
[R_1_,R_2_,R_3_=*t-*Bu]					
2,6-(1-methoxyethyl)Phenyl (**16**)		Ar*SeOTf electrophile	57	Bu_3_SnH/AIBN	57
[R_1_,R_3_=CH(OCH_3_)CH_3_; R_2_=H]					
Benzyl (**17**)	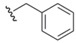	(BnSe)_2_ electrophile	44	Br_2_/NH_2_NH_2_	44,58
		BnSeCH_2_Br electrophile	58		

Note: See Table 2 for additional Se-protecting groups specific for selenocysteine.

Certain functionality commented on in this review could easily be considered "intermediate architecture" as opposed to an authentic protectant due to their apparent lack of strong protection profile or perhaps even a propensity to activate the Se functionality via the installation of an umpolung instead of attenuating its reactivity as is traditionally expected from a protecting group. All Se functionalization described in this review, however, does provide an avenue into synthetic protocol afforded to the selenol functionality not achievable in its native architecture.

## 2. Discussion

### 2.1. Heteroatom-Containing Se-Protection

**Figure 3:**
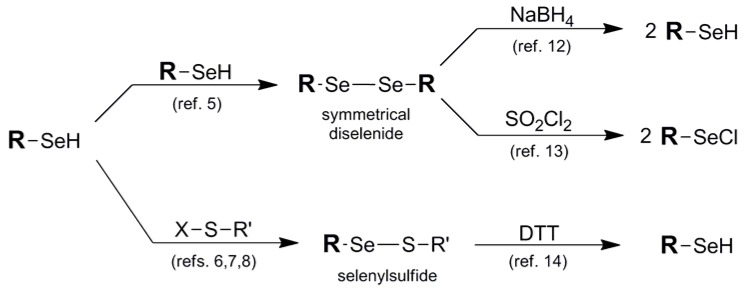
Synthetic routes into diselenide and disulfide protection schemes and their deprotection pathways to release their corresponding selenol functions.

Ironically, one of the most effective protection protocols for the selenol-containing system is its union with another molecule of itself in the form of a symmetrical diselenide motif **1**, or paired with a thiol "cap" in the form of a selenylsulfide **2**. Most commercially-available selenol-bearing compounds are offered as their corresponding symmetrical diselenides unless they are previously protected in another fashion. This is due to the high propensity of the selenol functionality to spontaneously oxidize to its corresponding diselenide under ambient conditions. Analogously, installation of selenylsulfide protection results from the covalent attachment of an asymmetric thiol small molecule to deaden the reactivity of the original selenol [4]. Diselenide and selenylsulfide pairing is an oxidatively favorable process, particularly involving the union of the higher chalcogens. As such, formation of the symmetrical diselenide protection is a facile or spontaneous process [5] in most selenol systems while selenylsulfide protection framework must be installed iteratively typically *via* reaction of the selenol with an electrophilic sulfur partner in order to avoid disproportionation (Figure 3) [6,7,8]. Alternatively, in some cases the selenium partner has acted as the electrophile with endogenous added thiol as the nucleophilic partner in the iterative design of the selenylsulfide system [9,10].

Regeneration of the original selenol function in diselenide and selenylsulfide blocking protocols typically requires quite different reducing conditions due to the great difference in redox potential between diselenides and selenylsulfides [11]. Due to the extreme durability of the diselenide function, regeneration of the original selenol typically requires comparatively forcing reduction conditions such as borohydride [12]. Alternatively, to liberate the selenium atom as an electrophilic functionality, treatment with sulfuryl chloride affords the selenyl chloride [13], poised for further potential derivatization. Deprotection of the selenylsulfide moiety, by comparison, is primarily carried out reductively using DTT or analogous thiol reduction conditions [14].

**Figure 4:**
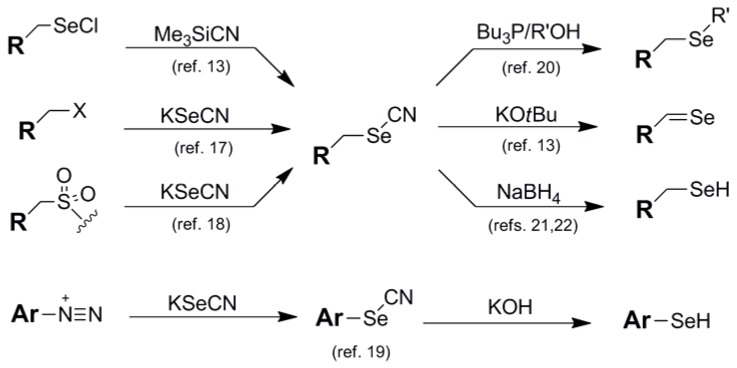
Synthetic routes into Cyanate protection schemes and their deprotection pathways to release their corresponding free selenol and other selenium-containing functions.

Cyano-containing blocking groups have played a part in a large number of diverse Se protection schemes, and are mentioned here in order of increasing stability of the intermediate. The thiocyanate (SCN) functionality **3** has limited mention in the literature as a stand-alone Se blocking motif due to its inadequate stability as a selenyl substituent [15]. Its primary utility has been as an electrophilic selenium umpolung-inducing function in tandem with enolate nucleophiles for direct α-selenylations in propanone-based test systems [16]. The standard cyano (CN) group **4** is a commonly-used Se blocking motif which exhibits modest interim stability. As such, it has been used both as a standard blocking protocol as well as an umpolung-inducing design to aid in the direct electrophilic transfer of selenium functionality. Installation of the cyano group is typically carried out via direct insertion of KSeCN nucleophile onto various electrophiles, including alkyl halides [17], sulfamidates, [18], and aryl diazonium species [19]. More exotic means of SeCN introduction have been carried out by Back and coworkers using Me_3_SiCN/RSeCl partners [13]. Beyond their use as a standard protective element for the selenol function, the umpolung-inducing abilities of selenocyanates can be utilized in their direct conversion to selenylsulfides [9] as well as their use as intermediates in their conversion to selenides (selenoethers) via reaction with primary alcohols and Bu_3_P [20] and oxidation to selones via treatment with KOtBu [13] (Figure 4). Actual removal of cyano protection to regenerate the native selenol can proceed under diverse sets of conditions. Aryl selenols can be regenerated from their corresponding selenocyatates by treatment with KOH [19] while alkyl selenocyanates are typically deprotected via borohydride reduction [21,22].

**Figure 5:**
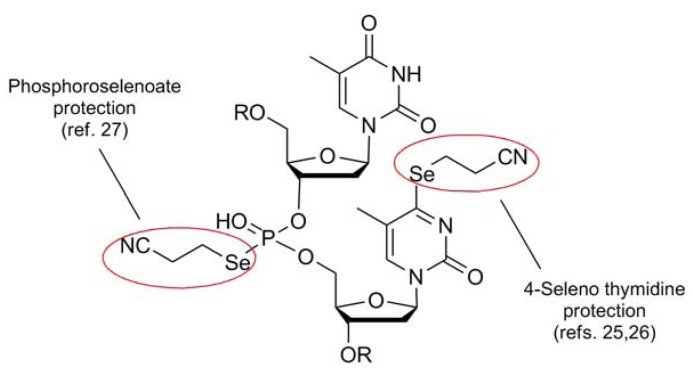
Use of 2-Cyanoethyl protection in the synthesis of Se-containing nucleotide analogs.

The cyanoethyl blocking group **5** of Huang [23] is the only member of the cyano series which behaves like a stand-alone protection protocol due to its high degree of stability coupled with its ability to effectively mask the reactivity of its corresponding selenol function. It functions similarly to the same architecture found in the analogous thiol protection scheme [24], and shares much of the same deprotection protocol as the selenium analog. Perhaps due to the fact that this protecting group has been developed and used by virtually one research group, only one type of Se-containing nucleotide system has made use of the 2-cyanoethyl moiety as a protection scheme (Figure 5). 

Typically introduced as the 2-cyanoethylselenide nucleophile, delivery of the selenium atom occurs concomitantly with the protecting group module itself, displacing either a triazolide [25] or sulfonate [26] electrophile to install selenium functionality at the 4-position of thymidine or the 6-position of guanine respectively. Recently, the group of Yan has utilized this protection scheme for the protection of selenated intermediates in their synthesis of oligonucleotide phosphoroselenoates [27]. Noteworthy in this case was that Se-incorporation was effected using a 2-cyanoethylselenyl phthalimide (*vide infra*) for the installation of the protected selenium as an electrophilic transfer agent. 

Deprotection of 2-cyanoethyl protection to regenerate free selenium has normally used the same conditions as for the protected sulfur analog [24]. Basic conditions of K_2_CO_3_/MeOH cleanly removes this blocking group to afford the free selenium moiety [23,25,26]. Alternatively, DBU/DCM has been utilized for the deprotection of systems which require a non-protic matrix for removal [27].

**Figure 6:**
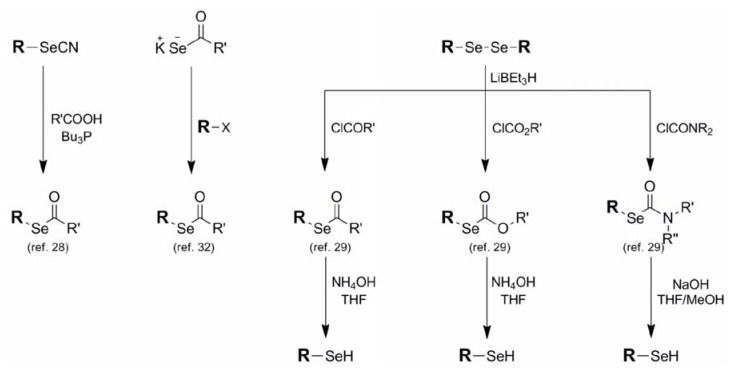
Synthesis and deprotection conditions for selenoacetates, selenocarbonates, and selenocarbamates.

Selenoacetates **6**, carbonates **7**, and carbamates **8** belong to a related family in which the selenol moiety is protected as its corresponding carbonyl conjugate. Although selenoalkylate systems have been known and studied for some time [28], Tour and coworkers published a very complete account in 1998 which methodically illustrated the syntheses of all of these systems from corresponding selenols as well as the specific deprotection profile of each structural type [29,30]. The most common method for the formation of these protection schemes is through the reaction of *in-situ*-derived selenoates with the appropriate acetyl chloride, chloroformate, or carbamyl chloride to afford the corresponding acetate, carbonate, and carbamate respectively (Figure 6). Similarly, *in-situ*-derived selenoates condensing with less reactive electrophiles such as esters [31] has been reported. In a noteworthy reversal of reactive partners, selenoacetate formation has been reported between potassium selenoacetate and various alkyl halides [32]. Grieco used the unusual combination of selenocyanates and carboxylic acids under phosphine-mediated conditions to afford a variety of selenoalkanoates, albeit in modest yields [28]. 

These protection schemes, although classically referred to as "activated" esters, have acceptable blocking abilities in non-alkaline environments. Removal of the alkanoate, carbonate, and carbamate protection to regenerate the corresponding selenol typically requires basic conditions of varying strength depending on the protective functionality. The alkylate and carbonate functionalities typically require treatment with NH_4_OH to effect removal while the carbamate moiety, being more robust, requires more forcing NaOH conditions for its deprotection [29,32]. 

**Figure 7:**
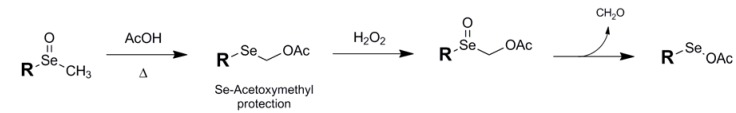
Formation of Se-Acetoxymethyl protection via Pummerer rearrangement and deprotection using peroxide.

Occasionally an unexpected transformation can be serendipitous in that the product can have a useful application beyond what was expected. This appears to have been the case with the Se-acetoxymethyl conjugate **9** of Sonoda and coworkers which has strong potential application as a selenium protectant [33]. The formation of this Se protectant is achived via the Pummerer reaction of a methyl selenoxide with acetic acid (Fig. 7). 

Although robust in its classic form, the acetoxymethyl framework becomes unstable upon re-oxidation of the selenium with H_2_O_2_, spontaneously extruding formaldehyde to form the fragile selenooxyacetate which has the potential to be easily reduced to the free selenol, although the authors chose not to illustrate this pathway. The single reference to this protection scheme in the literature instead traps the reactive selenooxyacetate intermediate with exogenous alkene to yield acetoxyselenated products, and there is no further mention of any protectant capability of the Pummerer-induced precursor.

**Figure 8:**
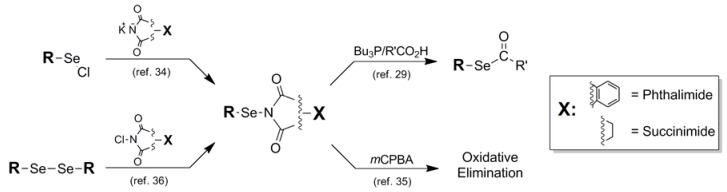
Synthetic routes into Phthalimide and Succinimide protection schemes and their routes for removal.

As previously stated, certain types of functionalized selenium serve less as a blocking protocol and more as an intermediate in a reaction sequence. This is the case with the phthalimide **10** and succinimide **11** Se protection motifs (Fig. 8). As selenium-containing reactive intermediates go, the Se-N-conjugated phthalimide and succinimide constructs are fairly stable crystalline solids which can be stored intact for reasonable lengths of time [34]. The most prevalent manner in which this collective functionality has been utilized in the literature has been for electrophilic addition of selenyl functionality to alkenes in the presence of exogenous or endogenous nucleophile, spotlighting their dual functions as protecting groups and reactive electrophiles [34,35,36]. Introduction methods differ for the installation of this family of blocking protocol as Se protection. The earliest reference by Nicolaou for the manufacture of both phthalimide and succinimide Se protection describes the condensation of potassium phthalimide/succinimide nucleophile onto a selenyl chloride electrophilic partner [34]. Alternatively, Sharpless synthesized selenyl succinimides using a disproportionation reaction between *N-chloro*succinimide and aryl diselenides [36]. This mixture was carried out with added alkene *in-situ *to afford arylselenated pinene intermediates which were ultimately shown to be autocatalytic in the formation of allylic chlorination products from pinene-based systems. 

Since the phthalimide and succinimide Se-blocked systems are used primarily as reactive intermediates as opposed to classically-functioning protecting groups, there is no reference to a conventional set of deprotection conditions to afford the native selenol functionality. Instead, any mention of removal of the phthalimide or succinimide motifs is concurrent with selenium elimination in the final product. In what will become a diagnostic and representative example in many further accounts of selenium protection in this review, Marquez subjects the oxyselenation products of various alkenes to oxidative elimination using *m*CPBA as oxidant [35]. A noteworthy transformation mediated by a phthalimide-conjugated selenol is its utilization by Grieco [37] in the synthesis of selenoalkylate and -arylate esters described previously as protecting groups in their own fashion [29]. Under phosphine-mediated conditions, arylselenophthalates were found to be smoothly converted to their corresponding selenoesters in the presence of added carboxylic acid reaction partner in very good yields. This stands in contrast to the similar previously-mentioned selenoalkylate synthesis in which a selenocyanate was used as the selenium delivery module under identical conditions [28]. The selenocyanate-mediated process gave, by comparison, much lower yield of selenoester than the selenophthalimide-mediated process.

### 2.2. Hydrocarbon-based Se-Protection

Up to this point, most of the previously-described selenium protecting groups have been structurally based upon heteroatom-containing functionality, with their respecting reactivities heavily dependent upon the presence of these non-carbon elements. What follows is a listing of strictly hydrocarbon-based alkyl and aryl protective architecture for the selenol function. The simplest alkyl blocking moiety for selenium would be the methyl group **12**, possessing the dubious distinction of "permanent" selenium protection due to its seeming lack of removal conditions once installed. As the term "permanent" implies, methyl functionalization of a selenol is indeed meant to block unwanted reactivity. However, the regeneration of the blocked selenol is never a priority of the synthetic design in all references to methyl Se protection. Similar to prior Se-protection schemes, installation of the Se-methyl protectant can be achieved in one of two ways. From a previously-existing selenol, treatment with methyl iodide easily affords the requisite SeMe architecture [13,38]. Alternatively, methyl protection can be delivered concomitantly with the selenium functionality. Examples of this include Mortikov's reaction of an aryl lithiate with dimethyl diselenide [39] to install the selenium methyl-protected, as well as the method of Huang in his continuing evolution of selenium-functionalized oligonucleotides, using NaSeCH_3_ as exogenous nucleophile attacking an anhydrouridine electrophile [40]. 

In none of these referenced syntheses is there any attempt to deblock the methyl-functionalized selenium once it has been installed. Moreover, the Se-methylated status of the constructs mentioned here is actually secondary to the principal focus of the respective research goals stated in the publications. Indeed, Liotta utilized selenomethyl handles in β-dicarbonyl test systems to effect oxidative elimination of the entire selenium functionality in his syntheses of various corresponding Michael acceptor products [38]. This again cannot be considered a classical deprotection since the selenium atom is being completely removed concomitantly with the protective motif, as previously described in the aforementioned phthalimide-based protection schemes [35]. 

There is some mention in the literature, however, of forcing conditions which will remove methyl functionality from selenium, albeit affording a highly-reactive product compound. Renson and coworkers utilize molecular bromine to effect methyl removal from an aryl-methyl selenoether, affording a selenylbromide intermediate [41]. While in this case the selenium functionality was released as its corresponding selenyl bromide which was further reacted *in-situ* to effect intramolecular Se-N bond formation in ebselen-templated systems, it had potential for simple reduction to yield a free selenol. As previously shown, methyl selenides are precursors of the acetoxymethyl Se-protection scheme **9*** via* Pummerer rearrangement [33]. Although this transformation hasn't been accomplished in Se-methyl-protected systems *per se*, it would seem to offer promise as a conversion under less-forcing conditions which could ultimately result in regeneration of the native selenol. 

In a manner reminiscent of the 2-cyanoethyl blocking protocol of Huang [23], another example of a selenium blocking group whose manufacture and use is specific to a particular research group is the Se-allyl protection scheme **13** of Back and coworkers [42]. An architecture exclusive to selenol protection, the Se-allyl conjugation has been used by Back in 3-selenium-functionalized camphor-based systems [42,43]. Introduction of the allyl functionality is carried out in standard fashion *via* allylation of an *in-situ*-generated selenoate with allyl iodide. Once functionalized, further chemistry may be carried out on the molecule while leaving the selenium undisturbed. Deprotection can then be effected *via* treatment of the allyl-blocked selenol first with *m*CPBA followed by hydrazine. 

**Figure 9:**
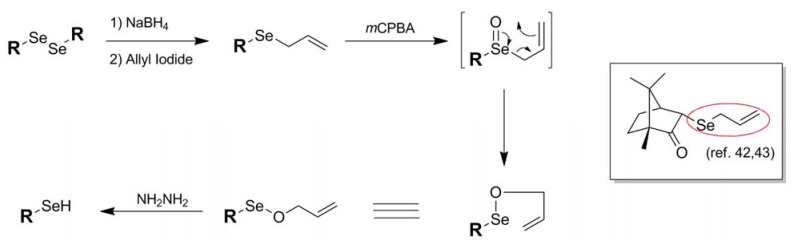
Installation and deprotection mechanism of Se-Allyl-based protection scheme.

Deprotection of the allyl functionality from selenium has its roots in the known use of allyl oxyselenium species to mediate chirality transfer through its natural rearrangement [44]. As illustrated in Figure 9, the oxidized Se-allyl species in the case of standard allyl deprotection spontaneously undergoes a [2,3]sigmatropic rearrangement to yield an Se-O-allyl species poised for reduction by added hydrazine. Once generated, the native selenol spontaneously forms a diselenide species in all of the systems studied. It is significant that, once oxidized, the selenium species undergoes rearrangement instead of oxidative elimination which is the typical outcome of oxidized selenoethers bearing β-alkyl hydrogens. It is uncertain in this case whether the [2,3]sigmatropic rearrangement is the preferred pathway because it is an energetically more favorable process or whether oxidative elimination is suppressed due to the strained architecture of the camphor-based substrates studied.

**Figure 10:**
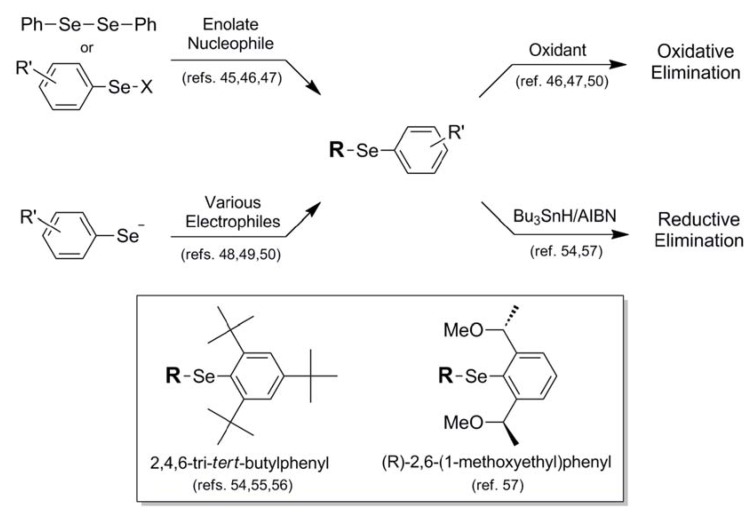
Synthetic routes into phenylselenyl protection schemes and their oxidative or reductive removal pathways.

When describing the "protective" nature of blocking groups for the selenol moiety, occasionally one encounters applications in which the blocking protocol is meant to be of a permanent nature [38]. Further, this particular functionalization is meant to prepare the selenium for complete removal from the system, sometimes exacting an additional transformation in the process. This is the case when describing Se-phenyl protection **14** and functionalized analogs **15** and **16**. In most literature accounts, Se-phenyl blocking protocol **14** is overwhelmingly a precursor for oxidative elimination, allowing the installation of an unsaturation into a molecular framework. Installation of the Se-phenyl functionalization proceeds by two (now somewhat familiar) general synthetic pathways, all involving delivery of the phenylselenyl moiety as a singular module. The most commonly utilized protocol is attack of an enolate nucleophile on a selenyl electrophile, either in the form of diphenyldiselenide [45] or phenylselenyl chloride [46,47]. Alternatively, The phenylselenyl component can act as nucleophile, delivered to various types of electrophiles such as allylic halides [48], Michael acceptors [49], and epoxides [50] (Figure 10). Once installed and functionalized, oxidative elimination can be carried out on the functionalized selenium using a wide variety of oxidants, including hydrogen peroxide [46], ozone [47], and sodium periodate [50]. A representative example with high synthetic merit is van der Donk's synthesis of dehydroalanine-containing peptides *via* oxidative elimination of phenyl-conjugated selenocysteine residues [51].

It is somewhat striking that in virtually all literature accounts there appears to be no fate for the Se-phenyl blocking protocol other than oxidative elimination. Since the phenyl architecture imparts great stability to the selenium atom, it would be of great synthetic importance to devise a methodology for its removal to regenerate the selenol functionality as a final synthetic step. It is noteworthy that in analogous sulfur-containing systems, phenyl thioethers can be cleaved back to their corresponding thiols either *via* electrolysis [52] or through the use of Pd(OAc)_2_/TBDMS-H [53]. It is unclear from proceedings in the literature whether these methods have been attempted for corresponding Se-phenyl systems. 

There are various Se-phenyl derivatives in the literature which bear auxiliary functionalization toward a specific end, although again the ultimate fate of the selenium atom is to be jettisoned *via* reductive elimination once its purpose has been completed. Toshimitsu and coworkers have found an enduring niche through their use of substituted Se-phenyl derivatives toward rather diverse functions. In a series of publications, the researchers describe the use of highly-sterically-protected 2,4,6-tri-*tert*-butylphenyl group **15** (Fig. 10) to maintain stereointegrity in episelenonium intermediates derived from β-selenoalcohols during carbon-carbon bond formation [54,55,56]. The steric bulk of this functionality also prevents unwanted selenophilic reactivity during the reaction sequence. In a later publication, Toshimitsu makes use of 2,6-chirally-substituted Se-phenyl functionality **16** to direct asymmetric carboselenation attack on various alkene substrates [57]. As is typically the case in the native phenyl-protection protocol examples, both of these substituted aryl moieties are ultimately jettisoned along with the selenium function itself, in these cases by reductive elimination using the Bu_3_SnH/AIBN reagent combination [54,57].

The benzyl group (Bzl) **17** has found limited use as a selenium protectant in non-peptidyl organic systems, for instance in Reich's synthesis of selenium-substituted bridged [2,2]paracyclophane systems [44]. The benzyl protection was installed with accompanying selenium *via* attack of an aryl lithiate on a benzyl diselenide electrophile. This selenated intermediate underwent Se-substitution exchange by deprotecting the benzyl moiety with a Br_2_/hydrazine combination to yield the free selenol which was subsequently air-oxidized to the corresponding diselenide. Reich also had an interest in the synthesis of benzyl-protected selenocysteine-containing systems through the unusual reaction sequence of treatment of protected glycine enolates with bromomethyl benzyl selenides to yield rudimentary Sec systems without regard for stereochemical purity [58]. Identical deprotection conditions (Br_2_/hydrazine) were utilized for Se deprotection of these constructs.

### 2.3. Selenocysteine Se-Protection

In organoselenium chemistry, selenocysteine (Sec, U) plays a large and important role as the most prevalent source of bioorganic selenium as well as the major representation of any selenium-containing biomolecule. As such, it is important to highlight this compound from a synthetic standpoint in order to be current with the many pathways which lead to its construction. Given that the method in which Sec is chemically incorporated into synthetic peptides and proteins is overwhelmingly *via* SPPS, the amino acid derivative which is used as the corresponding peptide building block must be orthogonally protected at its α-nitrogen as well as at its reactive selenol function. Standard current practice for α-nitrogen protection is almost exclusively tert-butoxycarbonyl (Boc) or 2-fluorenylmethyloxycarbonyl (Fmoc) depending on whether acidic or basic conditions are utilized to effect ^α^*N*-deprotection respectively to continue building the peptide sequence. The selenol protectant, meanwhile, must be stable to the conditions used for ^α^*N*-protection. Table 2 illustrates the known orthogonal Se protection schemes for Sec with simultaneous Boc, Fmoc, or benzyloxycarbonyl (Z) ^α^N protection. 

**Table 2:** Known selenocysteine protection schemes.

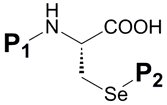					
**P_1_**	**P_2_**	**Method of Introduction**	**Ref**	**P_2_ Deprotection** **Conditions**	**Ref**
**Z**	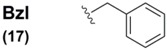	BnSe^-^ nucleophile	61	Na/NH_3_	59,60
**Boc**		--	--	--	--
**Boc**	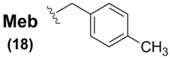	Meb-Br electrophile	63	HF	63,64
		MebSe^-^ nucleophile	64		
**Z**	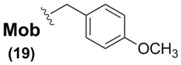	Mob-Cl electrophile	65	TFMSA/TFA	65
**Boc**		Mob-Cl electrophile	66,69	TMSBr/TFA	66,69
**Fmoc**		Mob-Cl electrophile	67	I_2_	67,68
				DMSO/TFA	67
		MobSe^-^ nucleophile	68		
				DTNP/TFA	70
**Boc**	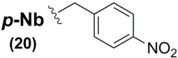	*p*Nb-Br electrophile	72	Zn, then I_2_	72
				SnCl_2_, then I_2_	72
**Boc**	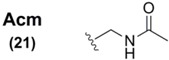	Acetamidomethanol/H^+^	72	I_2_	72

Interestingly, the vast majority of all known Sec Se protection schemes are structurally based upon the benzyl functionality, bearing diversified architecture at the *para* position on the phenyl ring (Table 2). The benzyl (Bzl) group **17** was the original standard Se protection protocol for the Sec sidechain. Used almost exclusively in tandem with the Z ^α^*N*-protection, benzyl blocked D/L Sec was used by Walter in early solution syntheses of oxytocin and deaminooxytocin [59,60] as well as other peptide systems [61]. Overwhelmingly, literature methods describing the removal of this blocking motif all involve the decidedly harsh treatment of the completed peptide with sodium in liquid ammonia. 

With the advent of Solid Phase Peptide Synthesis, Z ^α^*N*-protection became obsolete in favor of the aformentioned Boc and Fmoc protocols. In the case of the structural evolution of the Sec SPPS derivative, Bzl sidechain Se protection similarly fell quickly out of favor. Indeed, there is only one mention in the literature of a Bzl Se-protected Sec derivative bearing standard (Boc) ^α^*N*-protection [62], and this reference only describes the construction of the derivative, not its use in SPPS. This is likely due to the discovery and utilization of more labile benzyl-templated Se-protection protocol for Sec which didn't require such harsh conditions to effect their removal. 

The methylbenzyl (Meb) group **18** and methoxybenzyl (Mob) group **19** have found a considerable niche as the most enduring sidechain protectants for Sec, representing the only current Sec protectants in use today, with Sec(Mob) being the only Se-protection commercially-available. Known examples of Sec(Meb) protection is currently paired solely with accompanying Boc ^α^*N*-protection, and has been successfully applied to the synthesis of widely varying Sec-containing peptide systems [63,64]. Since the standard deprotection vector for Boc-derived peptide systems is *via* HF treatment, it is perhaps understandable that this is also the only method discussed in the literature for Sec(Meb) deprotection [63,64]. 

Sec(Mob) protection, in addition to being the only commercially-available Sec sidechain protectant, is by far the most widely used Se blocking protocol for Sec derivatives used in SPPS. It has been used in tandem with all three ^α^*N*-protection schemes (Z [65], Boc [66], and Fmoc [67,68]) in widely varying Sec-containing peptide syntheses. Once incorporated into its corresponding peptide systems, Sec(Mob) can then be deprotected using a variety of approaches. Due to the electron-releasing qualities of the *p*-methoxy group on the Mob architecture, the range of deprotection conditions can vary from the exceedingly harsh environments of TFMSA [65], TMSBr [66,69], and molecular iodine [68] to the more gentle and benign conditions of DMSO in TFA [67]. In a particularly gentle yet effective protocol, the group of Hondal showed that Sec(Mob)-containing peptides could be easily deprotected by treatment with substoichiometric quantities of 2,2'-dithiobis(5-nitropyridine) (DTNP) in TFA within one hour [70]. In further studies, these mild conditions have been found to be effective in the deprotection of Sec(Meb)- and Sec(Bzl)-containing peptides as well [71]. 

**Figure 11:**
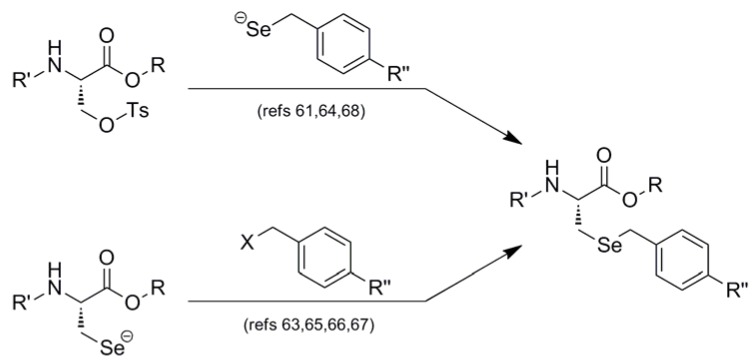
Dual synthetic routes into benzyl-templated Sec systems.

Interestingly, all synthetic approaches toward benzyl-templated selenocysteine systems adopt one of two synthetic vectors which have become familiar over the course of this review (Figure 11). In the first pathway, the benzyl-templated selenium atom is delivered onto a tosylated serine electrophile [61,64,68], introducing the selenium separately from the remainder of the amino acid module. 

Alternatively, another (perhaps more traditional) approach involves attack of a Sec Se nucleophile onto a benzyl-type halide [63,65,66,67] to afford the identical protected Sec construct. Indeed, this latter approach has been used in Seebach's synthesis of the novel Mob-protected β^3^-homoselenocysteine derivatives, allowing its iterative incorporation into various peptide systems [69].

As previously mentioned, the paucity of Sec sidechain protection examples in the literature is striking, especially considering that virtually all of the known protection protocol is based upon one type of architecture (the benzyl motif). Recently, however, new examples of Sec protection have emerged as viable and effective models in the construction of new Sec SPPS derivatives. The group of Alewood recently reported the synthesis and use of Sec derivatives bearing *p*-nitrobenzyl (*p*-Nb) **20** and acetamidomethyl (Acm) **21** sidechain protecting groups, illustrating their use in the synthesis of model peptides as well as highlighting the vectors of deprotection of each blocking moiety [72]. The researchers showed the *p*-Nb group to have a "reductive" vector of orthogonality in its deprotection profile when compared against other benzyl-templated Sec protection protocol. Using either Zn/AcOH or SnCl_2_-mediated conditions, the electron-attracting *p*-nitro group reduced to a strongly electron-releasing *p*-amino intermediate, allowing its facile removal with concomitant diselenide formation when treated with I_2_. 

The Acm group, by comparison, was shown to be similarly stable to acidic conditions (indeed, the conditions under which it was installed onto the Sec derivative were AcmOH/HCl) [72]. However, standard treatment with I_2_ effected the dual purpose of deprotection and diselenide formation in similar fashion to its analogous deprotection profile when it is used as a sidechain protectant for SPPS cysteine derivatives [73]. It is noteworthy to recognize that the aforementioned acetoxymethyl Se protectant **9** could be considered the oxygenated isoform of the Acm group, with possible potential for use as a Sec protectant in SPPS. Indeed, many of the previously-mentioned Se protecting groups have similar unexplored potential for placement into Sec derivatives for SPPS.

## 3. Conclusions

In striking contrast with the abundance of thiol protection noted in the literature, the corresponding scarcity of analogous protection for sulfur's chalcogen cousin selenium illustrates an interesting disparity in number and diversity of existing architecture. While it is certainly true that thiolate sulfur is more predominant in organic systems than corresponding selenol appearance, this disparity alone does not seem to address the scope of population gap in relative avenues for protection protocol. If anything, there exists incredible untapped synthetic potential for the exploration and design of new Se blocking architecture, either based upon the transfer of existing thiol protection vectors to corresponding Se systems or from the use of established (organic) Se protecting groups in selenocysteine sidechain protection.

## References

[1] Gladyshev V.N., Jeang K.T., Stadtman T.C. (1996). Selenocysteine, identified as the penultimate C-terminal residue in human T-cell thioredoxin reductase, corresponds to TGA in the human placental gene. Proc. Natl. Acad. Sci. USA.

[2] Epp O., Ladensteine R., Wendel A. (1983). The refined structure of the selenoenzyme glutathione peroxidase at 0.2-nm resolution. Eur. J. Biochem..

[3] Wuts P.G.M., Greene T.W. (2007). Protection for the Thiol Group. Protective Groups in Organic Synthesis.

[4] Prigol M., Wilhelm E.A., Schneider C.C., Nogueira C.W. (2008). Protective effect of unsymmetrical dichalcogenide, a novel antioxident agent, *in vitro* and an *in vivo *model of brain oxidative damage. Chem. Biol. Interact..

[5] Nauser T., Dockheer S., Kissner R., Koppenol W.H. (2006). Catalysis of electron transfer by selenocysteine. Biochemistry.

[6] Schneider C.C., Godoi B, Prigol M., Nogueira C.W., Zeni G. (2007). Highly stereoselective one-pot procedure to prepare unsymmetrical bis- and tris-chalcogenide alkenes via addition of chalcogens to alkynes. Organometallics.

[7] Zeni G.

[8] Flemer S., Lacey B.M., Hondal R.J. (2007). Synthesis of peptide substrates for mammalian thioredoxin reductase. J. Pep. Sci..

[9] Clark E.R., Al-Turaihi M.A.S. (1977). The reaction of *o*-nitro- and *p*-nitro-phenyl selenocyanates with arylthiols. J. Organometallic. Chem..

[10] Baldwin J.E., Haber S.B., Kitchin J. (1973). Dehydropeptides related to β-lactam antibiotics: a scheme for the biosynthesis of penicillins and cephalosporins. J. Chem. Soc. Chem. Comm..

[11] Besse D., Budisa N., Karnbrock W., Minks C., Musiol H.J., Pegoraro S., Siedler F., Weyher E., Moroder L. (1997). Chalcogen-analogs of amino acids: Their use in X-ray crystallographic and folding studies of peptides and proteins. Biol. Chem..

[12] Hondal R.J., Nilsson B.L., Raines R.T. (2001). Selenocysteine in native chemical ligation and expressed protein ligation. J. Am. Chem. Soc..

[13] Back T.G., Dyck B.P., Parvez M. (1995). 1,3-diselenetanes and 1,3-dithietanes derived from camphor. Formation, structure, stereochemistry, and oxidation to selenoxide and sulfoxide products. J. Org. Chem..

[14] Nogueira C.W., Rocha J.B.T. (2010). Diphenyl diselenide; a Janus-faced molecule. J. Braz. Chem. Soc..

[15] Parr W.J.E., Crafts R.C. (1981). The electrophilic addition of selenenyl thiocyanates to olefins. Tetrahedron Lett..

[16] Rheinboldt H., Perrier M. (1950). Thiocyanates d'acides sélénéniques aromatiques. II. Condensation avec l'acétone.

[17] van Ende D., Krief A. (1975). Stereoselective isomerisations of disubstituted olefins via seleniranes and thiiranes (1). Tetrahedron Lett..

[18] Baig N.B.R., Chandrakala R.N., Sudhir V.S., Chandrasekaran S. (2010). Synthesis of unnatural selenocystines and β-aminosiselenides via regioselective ring-opening of sulfamaidates using a sequential, one-pot, multistep strategy. J. Org. Chem..

[19] Yavuz S., Disli A., Yildirir Y., Turker L. (2005). The syntheses of some novel (naphthanen-1-yl-selenyl)acetic acid derivatives. Molecules.

[20] Grieco P.A., Gilman S., Nishizawa M. (1976). Organoselenium chemistry. A facile one-step synthesis of alkyl aryl selenides from alcohols. J. Org. Chem..

[21] Muller J., Terfort A. (2006). Synthesis of pure aromatic, aliphatic, and araliphatic diselenides. Inorg. Chim. Acta.

[22] Ie Y., Hirose T., Yao A., Yamada T., Takagi N., Kawai M., Aso Y. (2009). Synthesis of tripodal anchor units beariung selenium functional groups and their adsorptoin behavior on gold. Phys. Chem. Chem. Phys..

[23] Logan G., Igunbor C., Chen G.X., Davis H., Simon A., Salon J., Huang Z. (2006). A simple strategy for incorporation, protection, and deprotection of selenium functionality. Synlett.

[24] Ohtsuka Y., Oishi T. (1986). A synthetic approach to taxane diterpenes. A synthesis of the bicyclo[5.3.1]undecenone ring system. Tetrahedron Lett..

[25] Salon J., Sheng J., Jiang J., Chen G., Caton-Williams J., Huang Z. (2007). Oxygen replacement with selenium at the thymidine 4-position for the Se base pairing and crystal structure studies. J. Am. Chem. Soc..

[26] Salon J., Jiang J., Sheng J., Gerlits O.O., Huang Z. (2008). Derivatization of DNAs with selenium at 6-position of guanine for function and crystal structure studies. Nucleic Acids Res..

[27] Tram K., Wang X., Yan H. (2007). Facile synthesis of oligonucleotide phosphoroselenoates. Org. Lett..

[28] Grieco P.A., Yokoyama Y., Williams E. (1978). Aryl selenocyanates and aryl thiocyanates: reagents for the preparation of acivated esters. J. Org. Chem..

[29] Reinerth W.A., Tour J.M. (1997). Protecting groups for organoselenium compounds. J. Org. Chem..

[30] La Groia A., Feroci M., Inesi A., Rossi L. (2006). Electrochemical synthesis of selenocarbonates. Lett. Org. Chem..

[31] Maeda H., Tanabe T., Hotta K., Mizuno K. (2005). Synthesis of *Se*-arylmethyl selenoformates by reaction of aluminum arylmethaneselenoates with formates. Tetrahedron Lett..

[32] Balakumar A., Lysenko A.B., Carcel C., Malinovskii V.L., Gryko D.T., Schweikart K.K.H., Loewe R.S., Yasseri A.A., Liu Z., Bocian D.F., Lindsay J.S. (2003). Diverse redox-active molecules bearing O-, S-, or Se-terminated tethers for attachement to silicon in studies of molecular information storage. J. Org. Chem..

[33] Miyoshi N., Murai S., Sonoda N. (1977). Oxyselenation: reaction of acetoxymethyl methyl selenide with olefins in the presence of hydrogen peroxide. Tetrahedron Lett..

[34] Nicolaou K.C., Claremon D.A., Barnette W.E., Seitz S.P. (1979). N-Phenylselenophthalimide (N-PSP) and N-Phenylselenosuccinimide (N-PSS). Two versatile carriers of the phenylseleno group. Oxyselenation of olefins and a selenium-based macrolide synthesis. J. Am. Chem. Soc..

[35] Liu P.S., Marquez V.E., Kelley J.A., Driscoll J.S. (1980). Synthesis of 1,3-diazepin-2-one nucleosides as transition-state inhibitors of cytidine deaminase. J. Org. Chem..

[36] Hori T., Sharpless K.B. (1979). Conversion of allylic phenylselenides to the rearranged allylic chlorides by N-chlorosuccinimide. Mechanism of selenium-catalyzed allylic chlorination of β-pinene. J. Org. Chem..

[37] Grieco P.A., Jaw J.Y. (1981). N-Phenylselenophthalimide. A useful reagent for the facile transformation of (1) carboxylic acids into either selenol esters or amides and (2) alcohols into alkyl phenyl selenides. J. Org. Chem..

[38] Liotta D., Saindane M., Barnum C. (1981). Reactions involving selenium metal. 2. A general procedure for the preparation of unsaturated β-carbonyl compounds. Tetrahedron Lett..

[39] Gol'dfarb Y.L., Lifvinov L., Mortikov V.P., Yu V. (1979). Condensed heteroaromatic systems including a thiophene ring. 36. New complex-forming and chelate compounds of the benzothiophene series with selenium as the donor. Khim. Geterotsikl. Soedin..

[40] Du Q., Carrasco N., Teplova M., Wilds C.J., Egli M., Huang Z. (2001). Internal derivatization of oligonucleotides with selenium for X-ray crystalography using MAD. J. Am. Chem. Soc..

[41] Weber R., Renson M. (1979). Transformation of 3-benzisoselenazolinones to benz[β]selenophene derivatives. Bull. Soc. R. Sci. Liege..

[42] Back T.G., Dyck B.P. (1996). Asymmetric cyclization of unsaturated alcohols and carboxylic acids with camphor-based selenium electrophiles. Chem. Commun..

[43] Back T.G., Dyck B.P., Nan S. (1999). Asymmetric electrophilic methoxyselenylations and cyclizations with 3-camphorseleno derivatives. Tetrahedron.

[44] Reich H.J., Yelm K.E. (1991). Asymmetric induction in the oxidation of [2,2]paracyclophane-substituted selenides. Application of chirality transfer in the selenoxide [2,3] sigmatropic rearrangement. J. Org. Chem..

[45] Nishiyama Y., Koguma Y., Tanaka T., Umeda R. (2009). Cesium carbonate-catalyzed α-phenylchalcogenation of carbonyl compounds with diphenyl dichalcogenide. Molecules.

[46] Miyano M., Smith J.N., Dorn C.R. (1982). A synthesis of 11-homo-aldosterone. Tetrahedron.

[47] Zaidi J.H., Waring A.J. (1980). Synthesis of 3,4-dihydro-3,3,8a-trimethylnaphthalene-1,6(2*H*, 8a*H*)-dione, a 4-acylcyclohexa-2,5-dienone. J. Chem. Soc. Chem. Comm..

[48] Salmond W.G., Barta M.A., Cain A.M., Sobala M.C. (1977). Alternative modes of decomposition of allylic selenoxides diastereomeric at selenium. Tetrahedron Lett..

[49] Gilman H., Cason L.F. (1951). Some addition reactions of chalcones. II. The preparation of some χ-ketoselenides. J. Am. Chem. Soc..

[50] Stevens R.V., Albizati K.F. (1984). Synthetic approach to the amphilectane diterpenes: the use of nitriles as terminators of carbocation-olefin cyclizations. J. Org. Chem..

[51] Okeley N.M., Zhu Y., van der Donk W.A. (2000). Facile chemoselective synthesis of dehydroalanine-containing peptides. Org. Lett..

[52] Mairanovsky V.G. (1976). Electro-deprotection: electrochemical removal of protecting groups. Angew. Chem. Int. Ed. Engl..

[53] Chung M.K., Schlaf M. (2004). A catalytic synthesis of thiosilanes and silthianes: palladium nanoparticle-mediated cross-coupling of silanes with thio phenyl and thio vinyl ethers through selective carbon-sulfur bond activation. J. Am. Chem. Soc..

[54] Toshimitsu A., Nakano K., Mukai T., Tamao K. (1996). Steric protection of the selenium atom of the episelenonium ion intermediate to prevent both the racemization of the chiral carbon and the selenophilic attack of carbon nucleophiles. J. Am. Chem. Soc..

[55] Toshimitsu A., Terada M., Tamao K. (1997). Intramolecular cyclization reaction via a sterically protected episelenonium ion intermediate. Chem. Lett..

[56] Toshimitsu A., Hirosawa C., Nakano K., Mukai T., Tamao K. (1997). Stereospecific transformations of chiral compounds using anchimeric assistance of arylthio and arylseleno group. Phosphorus Sulfur Silicon.

[57] Okamoto K., Nishibayashi Y., Uemura S., Toshimitsu A. (2005). Asymmetric carboselenylation reaction of alkenes with aromatic compounds. Angew. Chem. Int. Ed..

[58] Reich H.J., Jasperse C.P., Renga J.M. (1986). Organoselenium chemistry. Alkylation of acid, ester, amide, and ketone enolates with bromomethyl benzyl selenide and sulfide: preparation of selenocysteine derivatives. J. Org. Chem..

[59] Walter R., du Vigneaud V. (1965). 6-hemi-L-selenocystine-oxytocin and 1-deamino-6-hemi-L-selenocystine-oxytocin, highly potent isologs of oxytocin and 1-deamino-oxytocin. J. Am. Chem. Soc..

[60] Walter R., Chan W.Y. (1967). Syntheses and pharmacological properties of selenium analogs of oxytocin and demaino-oxytocin. J. Am. Chem. Soc..

[61] Theodoropoulos D., Schwartz I.L., Walter R. (1967). Synthesis of selenium-containing peptides. Biochemistry.

[62] Soda K., Nobuyoshi E. (1992). Glutathione derivative and medicine containing the same as active ingredient. Jpn Patent.

[63] Oikawa T., Esaki N., Tanaka H., Soda K. (1991). Metalloselenonein, the selenium analogue of metallothionein: synthesis and characterization of its complex with copper ions. Proc. Natl. Acad. Sci. USA.

[64] Metanis N., Keinan E., Dawson P.E. (2006). Synthetic seleno-glutaredoxin 3 analogues and highly reducing oxidoreductases with enhanced catalytic efficiency. J. Am. Chem. Soc..

[65] Tamura T., Oikawa T., Ohtaka A., Fujii N., Esaki N., Soda K. (1993). Synthesis and characterization of the selenium analog of glutathione disulfide. Anal. Biochem..

[66] Casi G., Roelfes G., Hilvert D. (2008). Selenoglutaredoxin as a glutathione peroxidase mimic. Chembiochem.

[67] Koide T., Itoh H., Otaka A., Yasui H., Kuroda M., Esaki N., Soda K., Fujii N. (1993). Synthetic study on selenocysteine-containing peptides. Chem. Pharm. Bull..

[68] Gieselman M.D., Xie L., van der Donk W.A. (2001). Synthesis of a selenocysteine-containing peptide by native chemical ligation. Org. Lett..

[69] Flögel O., Casi G., Hilvert D., Seebach D. (2007). Preparation of the β^3^-homoselenocysteine derivatives Fmoc-β^3^hSec(PMB)-OH and Boc-β^3^hSec(PMB)-OH for solution and solid-phase-peptide synthesis and selenoligation. Helv. Chim. Acta.

[70] Harris K.M., Flemer Jr, Hondal R.J. (2007). Studies on deprotection of cysteine and selenocysteine side-chain protecting groups. J. Pep. Sci..

[71] Flemer S., Hondal R.J.

[72] Muttenthaler M., Ramos Y.G., Feytens D., de Araujo A.D., Alewood P.F. (2010). *p*-Nitrobenzyl protection for cysteine and selenocysteine: a more stable alternative to the acetamidomethyl group. Biopolymers.

[73] Veber D.F., Milkowski J.D., Varga S.L., Denkewalter R.G., Hirschmann R. (1971). Acetamidomethyl. A novel thiol protecting group for cysteine. J. Am. Chem. Soc..

